# Tumor-Secreted GRP78 Promotes the Establishment of a Pre-metastatic Niche in the Liver Microenvironment

**DOI:** 10.3389/fimmu.2020.584458

**Published:** 2020-09-29

**Authors:** Lu Chen, Hao Zheng, Xiang Yu, Lei Liu, Heli Li, Huifen Zhu, Zhihong Zhang, Ping Lei, Guanxin Shen

**Affiliations:** ^1^Department of Immunology, School of Basic Medicine, Tongji Medical College, Huazhong University of Science and Technology, Wuhan, China; ^2^Britton Chance Center for Biomedical Photonics, Wuhan National Laboratory for Optoelectronics, Huazhong University of Science and Technology, Wuhan, China; ^3^MoE Key Laboratory for Biomedical Photonics, School of Engineering Sciences, Huazhong University of Science and Technology, Wuhan, China

**Keywords:** tumor-secreted GRP78, liver pro-metastatic niche, dendritic cells, macrophages, natural killers, immunomodulation

## Abstract

The liver is an immunologically tolerant organ and a common site of distant metastasis for various cancers. The expression levels of glucose-regulated protein 78 (GRP78) have been associated with tumor malignancy. Secretory GRP78 (sGRP78) released from tumor cells contributes to the establishment of an immunosuppressive tumor microenvironment by regulating cytokine production in macrophages and dendritic cells (DCs). However, the role of sGRP78 on tumor cell colonization and metastasis in the liver remains unclear. Herein, we found that GRP78 was expressed at higher levels in the liver compared to other tissues and organs. We performed intravital imaging using a sGRP78-overexpressing breast cancer cell line (E0771) and found that sGRP78 interacted with dendritic cells (DCs) and F4/80^+^ macrophages in the liver. Importantly, sGRP78 overexpression inhibited DC activation and induced M2-like polarization in F4/80^+^ macrophages. Moreover, sGRP78 overexpression enhanced TGF-β production in the liver. In conclusion, sGRP78 promotes tumor cell colonization in the liver by remodeling the tumor microenvironment and promoting immune tolerance. The ability of sGRP78-targeting strategies to prevent or treat liver metastasis should be further examined.

## Introduction

Tumor metastasis remains the major cause of cancer-related deaths. The liver represents a common site of distant metastasis for various cancers, including melanoma, breast cancer, and colorectal cancer. The successful colonization of distant organs by circulating tumor cells (CTCs) is key for cancer metastasis (1), and the local microenvironment of these organs plays decisive roles in this process. Prior to cancer cell dissemination, the primary tumor secretes cytokines and vesicles, which create a pre-metastatic niche in secondary organs and metastatic sites. Myeloid-derived suppressor cells (MDSCs) and other immune-suppressive cells and secreted factors are essential for the establishment of the pre-metastatic niche. Upon establishment of a pre-metastatic niche, CTCs migrate to and colonize secondary organs. Some of the CTCs survive or become dormant until the microenvironment is suitable for the development of micrometastases and eventually macrometastases (1, 2).

Glucose-regulated protein 78 (GRP78) belongs to a group of highly conserved heat shock proteins (HSP) with important stress response functions (3–5). GRP78 is involved in the unfolded protein response (UPR) and endoplasmic reticulum (ER) stress response (5, 6), as well as in cell metabolism, hypoglycemia, hypoxia, acidosis, viral infections, and DNA damage repair (7). GRP78 is upregulated during ER stress; GRP78 translocates from the ER to the cell membrane (mGRP78) or is secreted as soluble GRP78 (sGRP78) (8). sGRP78 has long been recognized as a resolution-associated molecular pattern, facilitating inflammation resolution (9–11). GRP78 is expressed at higher levels in cancer tissues than in adjacent healthy tissues, and its expression levels have been associated with tumor malignancy (12, 13). Furthermore, GRP78 expression has been associated with cancer cell invasion and drug resistance, hindering the efficacy of anti-tumor treatments (14, 15). However, the role of GRP78 in the tumor microenvironment remains unclear. Rodvold et al. (16) reported a potential role of GRP78 in the activation of antigen-presenting cells (APCs) and subsequent innate and adaptive immune responses. Notably, GRP78-deficient macrophages demonstrated adapted UPR with upregulation of activating transcription factor (ATF)-4 and M2-polarization markers (17). We previously demonstrated that sGRP78 promoted B cell differentiation into IL-10-secreting CD19^hi^ regulatory B cells (18) and dendritic cell (DC) differentiation into regulatory DCs (DCreg) (9–11). Moreover, GRP78-treated DCs facilitated the differentiation of regulatory T cells (Tregs) (11). Hence, targeting GRP78 has emerged as a promising approach to enhance the effects of anti-tumor therapies (19–21). For instance, betulinic acid has been shown to induce apoptosis in breast cancer by binding to the ATPase domain of GRP78 (22). Furthermore, antibodies targeting GRP78 enhanced the efficacy of radiotherapy in human glioblastoma and non-small cell lung cancer cells (20). The anticancer effects of the apoptotic cyclic peptide BC71 have been attributed to its ability to inhibit mGRP78 (23). Besides, GRP78 has been shown to affect tumor progression and therapeutic response by modulating the functions of immune cells found in the tumor microenvironment (24).

The liver contains high numbers of natural killer (NK) cells (25), which play important roles in the immune responses against hepatocellular carcinoma and various other cancer types (26). Andre et al. (27) showed that blockade of the NK inhibitory receptor NKG2A enhanced anti-tumor immunity in mice and humans by enhancing the effector functions of NK and CD8^+^ T cells. The crosstalk between DC and NK is mediated by the phosphorylation of the signal transducer and activator of transcription 3 (STAT3) (28). Zhou et al. (29) demonstrated that TLR7/8 agonists enhanced the anti-tumor effects of NK cells in hepatocellular carcinoma by augmenting NK-DC crosstalk. Furthermore, DCs and MDSCs inhibited NK cell activation in a TGF-β-dependent manner (30, 31). TGF-β directly inhibited NK cell effector functions and reduced the levels of NKG2D on their cell surface (32, 33). Nevertheless, the role of tumor-secreted GRP78 on the ability of hepatic APCs to regulate the pro-metastatic activities of NK cells is understudied.

Herein, we show that GRP78 is highly expressed in the liver of tumor-free mice. To elucidate the relevance of sGRP78 in the hepatic metastatic niche, we established a sGRP78-overexpressing cell line, which was used to establish an experimental liver metastasis mouse model. Using this mouse model, we found that sGRP78 mediates the formation of a pro-metastatic niche, supporting the rationale of sGRP78-targeting as a liver metastasis prevention strategy.

## Materials and Methods

### Mice

Female C57BL/6 mice were purchased from Hunan SJA Laboratory Animal Co., Ltd (Changsha, Hunan, China). CX3CR1-GFP mice in a C57BL/6 background were purchased from the Jackson Laboratory; in these mice, EGFP is expressed in monocytes, DCs, NK cells, and brain microglia, under the control of the endogenous *Cx3cr1* locus. All experiments were performed with mice aged 6–8 weeks. Mice were bred and maintained in specific pathogen-free (SPF) conditions at the Animal Center of Wuhan National Laboratory for Optoelectronics. All procedures involving animals were conducted in compliance with protocols approved by the Hubei Provincial Animal Care and Use Committee of Huazhong University of Science and Technology.

### Cell Cultures

The E0771 cell line was kindly provided by Professor Rong Xiang (Nankai University, Tianjin, China) and was authenticated in Beijing Microread Genetics Co., Ltd. by STR analysis. The B16F10 cell line was purchased from the BO STER Company (Wuhan, China). E0771 cells were stably transfected with the PB transposon system (a gift from Dr. Xiaohui Wu, Fudan University, Shanghai, China) (34), which contained a *CMV* promoter-driven mCherry or mCherry-sGRP78 (GRP78 GeneBank No: NM_001163434.1) coding sequence, and named as E0771-mCherry/E0771-mCherry-sGRP78 cells. B16F10 cells were stably transfected with the PB transposon system, which contained the mCherry-sgGRP78, mCherry or mCherry-sGRP78 coding sequence (B16-mCherry-sgGRP78, B16-mCherry and B16-mCherry-sGRP78 cells). All cell lines were regularly tested for mycoplasma using the MycoProbe Mycoplasma Detection Kit (R&D Systems, Minneapolis, MN, United States). E0771 cells were cultured in DMEM containing 10% fetal bovine serum (FBS), 100 U/mL Sodium Pyruvate, 100 U/mL non-essential amino acids, and 100 U/mL penicillin-streptomycin. B16F10 cells were cultured in ROMI-1640 containing 10% FBS and 100 U/mL penicillin-streptomycin. Cells were maintained at 37°C in a 5% CO_2_ incubator (Thermo Fisher Scientific, United States).

### Protein Quantitation

Tissues and organs of C57BL/6 mice at 8 weeks were harvested and their mass was measured. Tissue samples with the same wet weight were lysed in NP-40 lysis buffer (5 μL/mg) containing a protease inhibitor cocktail (Sigma-Aldrich). Lysates were separated and stored at −80°C until further use. 1 × 10^6^ cells were seeded in the plates and cultured in serum-free culture media for 24 h. Then supernatants and tissue samples were assayed by ELISA using the BiP (C50B12) Rabbit mAb (CST). The purified GRP78 protein was used as the standard sample. Data were analyzed by Welch’s ANOVA.

### Cell Proliferation Assay

The 6-well plates were seeded with 10^4^ E0771 tumor cells on day 0, and then the cells were counted for 7 consecutive days. Data were analyzed by Welch’s ANOVA (versus E0771 group).

### Wound Healing Assay

The 6-well plates were seeded with 4 × 10^5^ E0771 tumor cells. After the cells adhere to the wall, the wound was scratched as the 0 h. And CCD photographs record the wound healing at 0 and 24 h.

### Liver Metastasis Model

The mice were anesthetized by intraperitoneal (i.p.) injection of a mixture of 10 mg/kg xylazine and 100 mg/kg ketamine hydrochloride (Sigma, St. Louis, MO, United States). During anesthesia, body temperature was maintained at 37°C using a warm plate (Thermo Plate, TOKAI HIT, Shizuoka-ken, Japan). Mouse spleens were exposed by a small upper abdomen incision, followed by injection of 1 × 10^6^ E0771 or 5 × 10^5^ B16F10 cells. Seven minutes later, half of the spleen containing the tumor cell injection site was resected. The hepatic metastatic burden was assessed on day 21 for E0771 and day 15 for B16F10 after tumor cell inoculation. Hematoxylin and eosin (H&E) stain was purchased from Servicebio Biotechnology (Wuhan, China) and slides were scanned using a Nikon Ni-E (Nikon, Minato, Tokyo, Japan). Images were acquired using the NIS-Elements software and analyzed using ImageJ. The metastatic burden was calculated by dividing the area occupied by metastatic foci (mm^2^) by the total surface liver area (mm^2^).

### Isolation of Intrahepatic Leukocytes

Female C57BL/6 mice (7–8 weeks old) were sacrificed by cervical dislocation. The liver was dissected into 1 mm pieces and digested using collagenase IV (Worthington) and DNAase II (Sigma) for 30 min at 37°C. The digested liver extracts were filtered through a 70 μm cell strainer and centrifuged at 500 × *g* for 5 min. The resulting cell pellet was resuspended in 10 mL 35% Percoll containing 100 U/mL heparin and centrifuged at 700 × *g* for 15 min at room temperature. The cell pellet containing leukocytes was collected and resuspended in 3 mL red blood cell lysis solution (155 mmol/L NH_4_CL, 10 mmol/L KHCO_3_, 1 mmol/L EDTA, 170 mmol/L Tris; pH 7.3). After incubation for 3 min at room temperature, cells were washed twice with RPMI 1640 containing 5% FBS.

### Flow Cytometry

Antibodies against CD45 (104), CD3 (17A2), NK1.1 (PK136), CD19 (6D5), CD69 (H1.2F3), CD4 (RM4-4), CD8 (53-6.7), Ki-67 (11F6), CD146 (ME-9F1), Ly6G (1A8), CD11b (M1/70), F4/80 (BM8), CD11c (N418), MHC-II (M5/114.15.2), CD86 (GL-1), CD80 (16-10A1), PD-1 (RMP1-14), and FoxP3 (MF-14) were purchased from BioLegend. The fixable viability dye eFluor506 was purchased from eBioscience. Liver cell suspensions were subjected to surface staining with fluorescently labeled antibodies according to the manufacturer’s instructions. Cell viability was assessed using the fixable viability dye eFluor506 (eBioscience). Subsequently, cells were permeabilized using the Transcription Factor Buffer Set (Biolegend) and stained for Ki-67, FoxP3. Cells were analyzed on a CytoFLEX flow cytometer (Beckman Coulter, United States). Flow cytometry data were analyzed using FlowJo software (FlowJo, Ashland, OR, United States).

### Immunofluorescence Analysis

For the immunofluorescence analysis, liver tissues were fixed in 4% paraformaldehyde for 12 h at 4°C and then dehydrated in 30% sucrose solution. The tissues were then frozen in OCT (Sakura, Torrance, CA, United States) compound and sectioned into 20 μm slices using a freezing microtome (Leica, Germany). OCT was removed by washing three times in PBS, and the sections were immunostained with Alexa Fluor 647 anti-mouse F4/80 (BioLegend, Clone: BM8, Catalog: 123122), Alexa Fluor 647 anti-mouse CD11c (BioLegend, Clone: N418, Catalog: 117312) or Alexa Fluor 647 anti-mouse NK1.1 (BioLegend, Clone: PK136, Catalog: 108720) at 1:200 dilution. All the sections were imaged with Olympus IX83 confocal microscope outfitted with an UltraVIEW VoX 3D live cell imaging system (PerkinElmer). Images were analyzed with Image J software (National Institutes of Health).

### Cytokine Quantitation

Livers were harvested and their mass was measured at days 4 and 7 after injection. Tissue samples were lysed in NP-40 lysis buffer (5 μL/mg) containing a protease inhibitor cocktail (Sigma-Aldrich). Lysates were separated and stored at −80°C until further use. Samples were assayed using the LEGENDplexTM Mouse Cytokine Panel array (BioLegend) according to the manufacturer’s instructions. Data were analyzed with Legendplex software (BioLegend). Mouse TGF-beta ELISA kit (DAKEWE) was used to detect TGF-β levels in the samples.

### Intravital Imaging

CX3CR1-GFP C57BL/6 mice (6–10 weeks old) were inoculated with 1 × 10^6^ E0771-mCherry or E0771-mCherry-sGRP78 cells (day 0). On days 4 and 7, mice were anesthetized by i.p. injection of a mixture of 10 mg/kg xylazine and 100 mg/kg ketamine hydrochloride (Sigma, St. Louis, MO, United States). Mice were maintained anesthesia with isoflurane inhalation [1.5–2% (v/v) isoflurane in O_2_] and placed within a custom-designed imaging box. Throughout the imaging process, mice were placed on a heating pad to maintain a body temperature of 37°C (Thermo Plate, TOKAI HIT, Shizuoka-ken, Japan). Intravital imaging was performed using an Olympus IX83 confocal microscope outfitted with an UltraVIEW VoX 3D live cell imaging system (PerkinElmer). Images were acquired using a 20 × /0.75 NA objective and Volocity 6.3 (PerkinElmer) software. Images were analyzed with Image J software (National Institutes of Health).

### Data Analysis

Intravital cell movement was assessed using Image-Pro Plus (Media Cybernetics, Inc., Rockville, MD; RRID:SCR-007369) or Imaris 7.6 (Bitplane AG, Switzerland; RRID:SCR-007370) software. The mean velocity, mean displacement, arrest coefficient, and confinement ratio were determined. The mean velocity was used to determine the migratory speed in μm/min. Cells with a mean velocity of less than 2 μm/min were defined as immotile. The mean displacement was used to determine the initial displacement of the cells. The arrest coefficient was calculated as the percentage of time that the instantaneous velocity of each cell was less than 2 μm/min, as previously described (35, 36). The confinement ratio was calculated as the ratio of the maximum displacement of a given cell to its path length within a given time. Linear fitting was performed on the plotted curves to determine whether the cells underwent random movement.

### Statistical Analysis

Statistical analysis was performed using GraphPad Prism 6 (GraphPad Software, CA, United States). A one-way ANOVA followed by a *post hoc* test was used for multiple group comparisons, and Student’s t-test (two-tailed) was used for comparisons of two groups. Survival data were analyzed using log-rank (Mantel-Cox) test. Values were expressed as mean ± standard error of the mean (SEM). Two-sided *P* < 0.05 was considered statistically significant (^∗^*P* ≤ 0.05, ^∗∗^*P* ≤ 0.01, ^∗∗∗^*P* < 0.001). The numbers of animals used in each experiment were indicated in the figure legends.

## Results

### Tumor-Secreted GRP78 Enhances Tumor Invasion and Liver Metastasis

**FIGURE 1:**
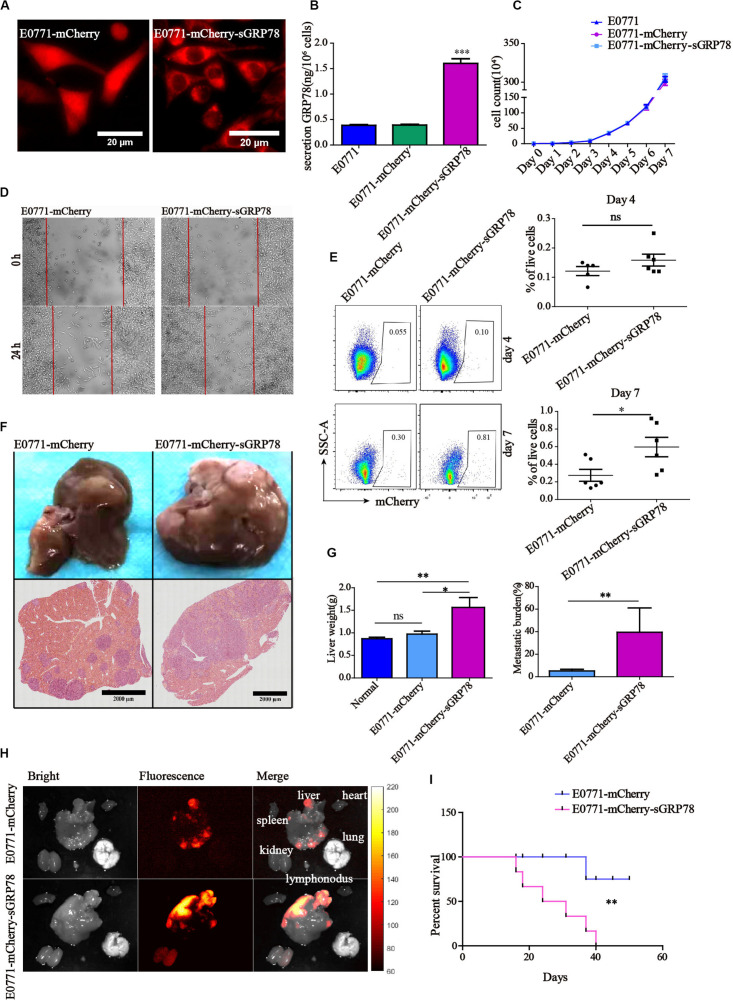
Tumor-secreted GRP78 promotes tumor invasion and liver metastasis. **(A)** sGRP78-overexpressing E0771-sGRP78-mCherry cells were identified by confocal microscopy. Scale bar, 20 μm. **(B)** The levels of sGRP78 in the cell culture supernatant were determined by ELISA. **(C)** Tumor growth curves. **(D)** The migration ability of tumor cells was assessed by wound healing assay. **(E)** Frequencies of mCherry-positive cells in the liver 4 and 7 days after cell inoculation. Representative flow cytometry dot plots (left) and percentage of positive cells (right) are shown. **(F)** Representative images of H&E-stained liver sections from mice sacrificed 20 days after tumor cell intrasplenic injection. Scale bar, 2,000 μm. **(G)** Quantification of liver weight (left panel) and metastatic burden (right panel) in livers (*n* = 5 mice per group). **(H)** Representative images of organ tumor burden 20 days after tumor cell intrasplenic injection. **(I)** Survival curves (*n* = 6). Error bars represent SEM. Ns, not significant; ^∗^*P* < 0.05; ^∗∗^*P* < 0.01; ^∗∗∗^*P* < 0.001. Statistical significance was determined by Student’s *t*-test (**E,G** right), by Welch’s ANOVA (**B,C,G** left) or by log-rank Mantel-Cox test **(I)**.

In this study, we established a sGRP78-overexpressing breast cancer cell line (E0771-mCherry-sGRP78). Confocal microscopy showed that, although mCherry primarily localized in the cytoplasm in E0771-mCherry-sGRP78 cells, it was evenly distributed in E0771-mCherry cells (Figure 1A). ELISA assay indicated that the level of sGRP78 in the supernatant of E0771-mCherry-sGRP78 cells was four times higher than that in E0771-mCherry or E0771 control cells (Figure 1B). Cell proliferation assay revealed that overexpression of sGRP78 did not affect the proliferation of E0771 cells *in vitro* (Figure 1C). To investigate the effect of sGRP78 overexpression on cancer cell migration, we performed a scratch wound healing assay. E0771-mCherry-sGRP78 cells were more migrative than E0771-mCherry control cells (Figure 1D).

We also found that the level of GRP78 in the supernatant (SN) of liver homogenate was two times higher than in the NS from spleen and lung (Supplementary Figure 1). Flow cytometry 4 days after cell inoculation indicated that the percentage of mCherry-positive cells that metastasized to the liver was 0.158% ± 0.020 in E0771-mCherry-sGRP78-bearing mice and 0.121% ± 0.015 in E0771-mCherry-bearing mice; this difference was no statistically significant (Figure 1E). Seven days after cell inoculation, activation of adaptive immune responses was observed in the liver, while the function of innate immune cells was less profound. In E0771-mCherry-sGRP78-injected mice, 0.595% of the living cells expressed mCherry, while the livers of E0771-mCherry-bearing mice contained approximately half as much (Figure 1E). Twenty days after splenic injection of tumor cells, we evaluated the effect of sGRP78 overexpression on liver metastasis by measuring the weight of the liver and histology (Figure 1F and Supplementary Figures 2A,B). Liver weight was higher nearly by a two-fold in E0771-mCherry-sGRP78-bearing mice compared with E0771-mCherry-bearing mice. Histology confirmed the higher metastatic burden in E0771-mCherry-sGRP78-bearing mice (Figure 1G). Additionally, widefield fluorescence imaging of different organs indicated that mCherry-expressing metastatic tumor cells were primarily found in the liver. Interestingly, weak mCherry signal was detected in the kidneys of E0771-mCherry-sGRP78-injected mice but not in E0771-mCherry-injected mice, suggesting that sGRP78 was excreted through the kidneys (Figure 1H). Notably, all E0771-mCherry-sGRP78-bearing mice died within 40 days after cell inoculation, while 75% of E0771-mCherry-bearing mice were still alive after 50 days (Figure 1I). The B16 cell lines had similar results, showing that GRP78 upregulation promoted more tumor cells (B16-mCherry-sGRP78) metastasizing to liver, while down-regulation of GRP78 (B16-mCherry-sgGRP78) significantly improved the survival ability of mice (Supplementary Figures 2C,D). These findings suggest that tumor-derived sGRP78 promotes tumor cell dissemination and colonization *in vivo*, accelerating liver metastasis.

### Tumor-Secreted GRP78 Induces Tolerogenic Phenotypes in Hepatic Dendritic Cells and Macrophages

**FIGURE 2:**
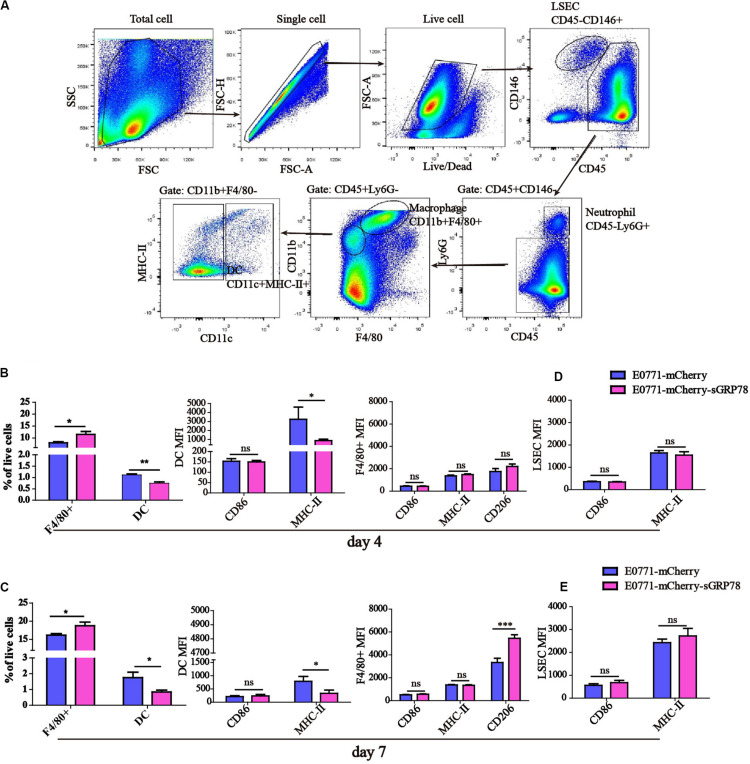
Tumor-secreted GRP78 induces tolerogenic phenotypes in hepatic monocytes/macrophages. **(A)** Gating strategy followed to distinguish the hepatic myeloid cells. Frequencies of hepatic F4/80^+^ macrophages and DC cells, as well as their expressions of CD86 and MHC-II four **(B)** and seven **(C)** days after tumor cell intrasplenic injection. Expression of CD86 and MHC-II on LSEC cells four **(D)** and seven **(E)** days after tumor cell intrasplenic injection. Graphs represent the mean ± SEM. Ns, not significant; ^∗^*P* < 0.05; ^∗∗^*P* < 0.01; ^∗∗∗^*P* < 0.001. Statistical significance was determined by Student’s *t*-test.

Next, we investigated the effects of sGRP78 overexpression on the function of hepatic APCs. Hepatic APCs mainly consist of DCs, Kupffer cells (KCs), macrophages, and liver sinusoidal endothelial cells (LSECs) (37, 38). We performed flow cytometry evaluating the frequencies of different hepatic APCs, as well as quantifying the expression levels of CD86 and MHC-II (Figure 2A). We found that 4 days after cell inoculation, the percentages of F4/80^+^ macrophages and DCs in the liver differ significantly between the two groups (F4/80^+^ macrophages were 11.48% in E0771-mCherry-sGRP78-bearing mice vs. 7.98% in E0771-mCherry-bearing mice; *P* < 0.05; DCs were 0.732% in E0771-mCherry-sGRP78-bearing mice vs. 1.136% in E0771-mCherry-bearing mice; *P* < 0.01; Figure 2B). This was also the case 7 days after cell inoculation; however, the level of macrophage recruitment was higher compared to 4 days after cell inoculation (Figure 2C). Additionally, 7 days after cell inoculation, the expression levels of the M2-type marker CD206 was profoundly higher in mice with tumor-secreted GRP78 compared with control mice (*P* < 0.001; Figure 2C). Although we found no difference in the levels of DC recruitment, the expression levels of MHC-II on DCs was significantly lower in the E0771-mCherry-sGRP78 group compared with control mice (*P* < 0.05; Figures 2B,C). sGRP78 did not affect the expression of CD86 or MHC-II on LSECs (Figures 2D,E). These results suggest that tumor-secreted GRP78 affects the hepatic immune microenvironment by inducing tolerogenic phenotypes in DC and F4/80^+^ macrophages, but not in LSEC.

### Tumor-Secreted GRP78 Binds to Hepatic DCs and F4/80^+^ Macrophages

**FIGURE 3:**
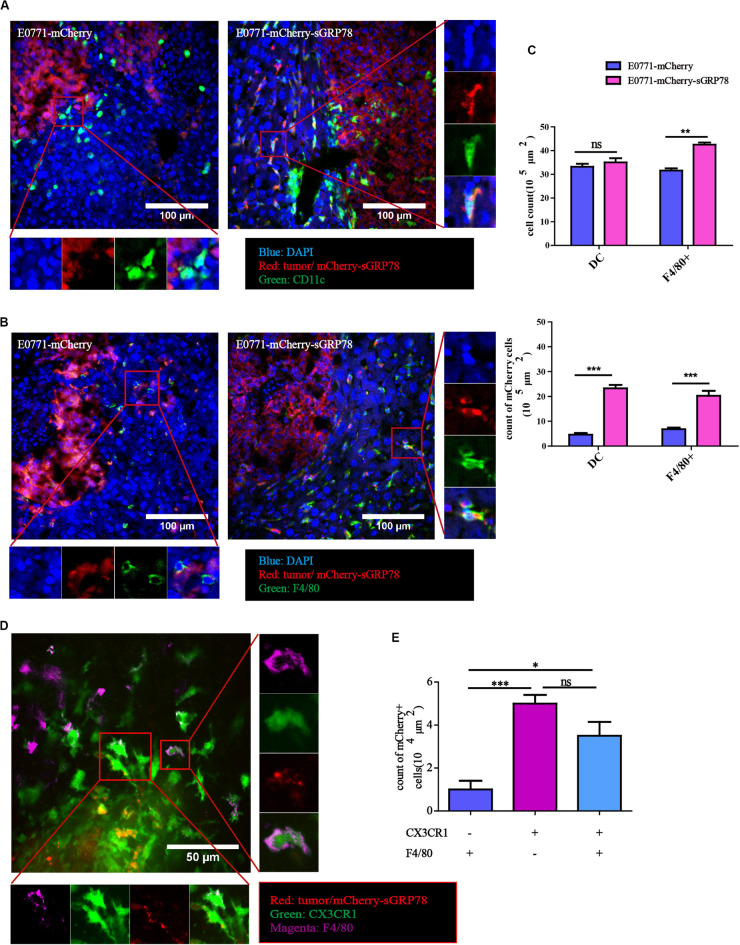
Tumor-secreted GRP78 interacts with hepatic DC and F4/80^+^ macrophages. Mice liver sections showing sGRP78 interacting with CD11c^+^ cells **(A)** and F4/80^+^ cells **(B)**. Scale bar, 100 μm. **(C)** Numbers of infiltrated DC cells and F4/80^+^ cells (upper), as well as of mCherry^+^ DC and F4/80^+^ cells (lower). sGRP78 was detected to interact with CX3CR1^+^ cells **(D)**. Scale bar, 50 μm. **(E)** Numbers of mCherry^+^ CX3CR1^+^ cells. Error bars represent SEM. Ns, not significant; ^∗^*P* < 0.05; ^∗∗^*P* < 0.01; ^∗∗∗^*P* < 0.001. Statistical significance was determined by Student’s *t*-test **(C)** or by Welch’s ANOVA **(E)**.

Immunofluorescence staining on liver sections from our experimental liver metastasis mouse model was performed to evaluate the ability of sGRP78 to bind to CD11c^+^ DCs (Figure 3A) and F4/80^+^ macrophages (Figure 3B). Tumor cells displayed strong mCherry signal, and in E0771-mCherry-sGRP78-bearing mice, sGRP78 was diffusely distributed and partially taken up by CD11c^+^ DC and F4/80^+^ macrophages. Although the density of F4/80^+^ macrophages in metastatic lesions was higher by a 1.3-fold in E0771-mCherry-sGRP78-bearing mice compared with control mice, the numbers of mCherry-positive CD11c^+^ DCs and F4/80^+^ macrophages were considerably higher in E0771-mCherry-sGRP78-bearing mice (5.4-fold for CD11c^+^ cells and 3-fold for F4/80^+^ cells; Figure 3C). These findings suggest that DCs and macrophages directly bind sGRP78.

CX3CR1 is expressed in various cells, including DCs and macrophages (39, 40). In our CX3CR1-GFP liver metastasis mouse model, tumor-secreted GRP78 was taken up by both F4/80^+^CX3CR1^+^ and F4/80^–^CX3CR1^+^ cells (Figure 3D). In theory, sGRP78 could also bind on KCs (F4/80^+^CX3CR1^–^). Compared to CX3CR1^–^F4/80^+^ cells, the numbers of mCherry-positive CX3CR1^+^F4/80^–^ and CX3CR1^+^F4/80^+^ cells were 5 times and 3.5 times higher, respectively (Figure 3E). These results confirm that CX3CR1 transgenic mice are a promising model for analyzing the behavior of DCs and macrophages during the establishment of the pre-metastatic niche in the liver.

### Intravital Imaging of Myeloid CX3CR1^+^ Cell Migration in Metastatic Lesions

**FIGURE 4:**
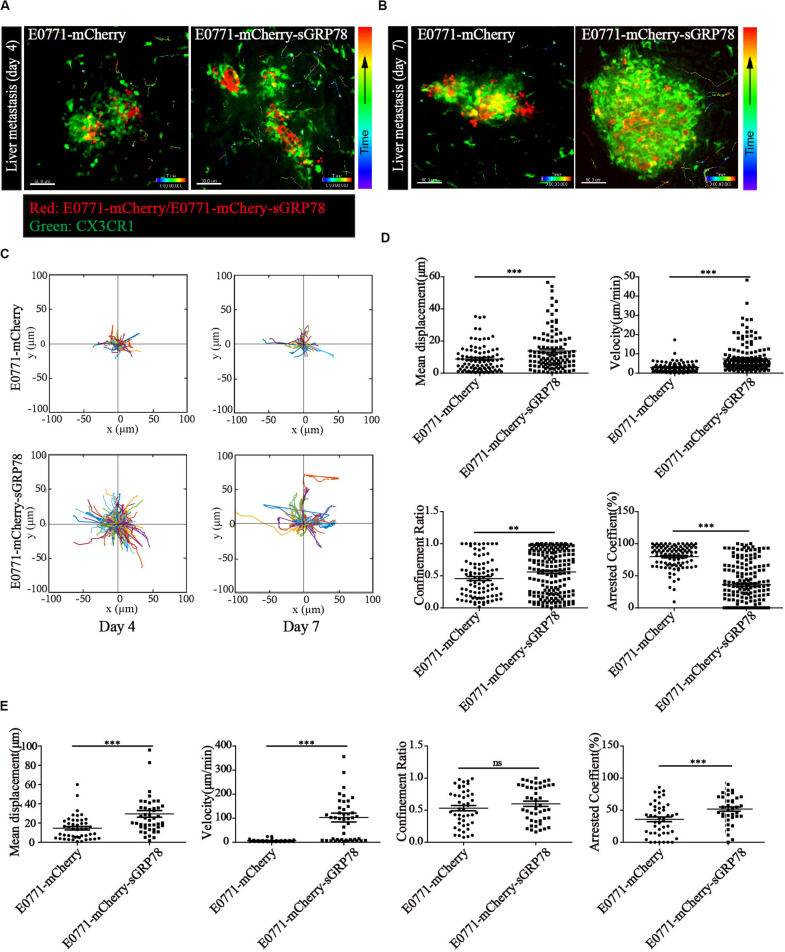
Tumor-secreted GRP78 promotes myeloid CX3CR1^+^ cell recruitment in metastatic lesions. *In vivo* imaging of the livers four **(A)** and seven **(B)** days after E0771-mCherry (left) and E0771-sGRP78-mCherry (right) injection, using confocal microscopy. Trajectories **(C)** and motion parameters of CX3CR1^+^ cells four **(D)** and seven **(E)** days after tumor cell intrasplenic injection. Each data point represents a single cell, and the red bars indicate mean values. ^∗∗^*P* < 0.01; ^∗∗∗^*P* < 0.001; ns: not significant. The data from 3–5 mice in two independent experiments were pooled. Statistical significance was determined by Student’s *t*-test.

To understand the effects of sGRP78 on the motility of hepatic myeloid DCs and macrophages within the tumor microenvironment, we performed intravital imaging using C57BL/6 CX3CR1-GFP mice. Intravital imaging was performed using confocal laser scanning microscopy (CLSM) (Figures 4A,B), followed by CX3CR1^+^ cell motility quantification (Figure 4C). Four days after tumor cell inoculation, the motility of CX3CR1^+^ cells in the E0771-mCherry-sGRP78-bearing mice was significantly increased. The 25-min mean displacement of CX3CR1^+^ cells was higher in the E0771-mCherry-sGRP78 group compared with the E0771-mCherry group (13.85 ± 1.177 μm vs. 8.759 ± 0.8735 μm; *P* < 0.001; Figure 4D). In the E0771-mCherry-sGRP78 group, CX3CR1^+^ cells migrated to the tumor periphery with a mean velocity of 7.350 ± 0.5245 μm/min (*n* = 175 cells). However, in E0771-mCherry-bearing mice, CX3CR1^+^ cells migrated with a mean velocity of 2.899 ± 0.2512 μm/min (*n* = 95 cells). Moreover, in E0771-mCherry-sGRP78-bearing mice, the migration trajectories were more confined (confinement ratio: 0.5610 ± 0.02504; *P* < 0.01) and the arrest coefficient (percentage of cell resting time) was lower compared with the E0771-mCherry group (36.14% ± 2.446 vs. 79.93% ± 1.858; Figure 4D).

Seven days after tumor cell inoculation, the CX3CR1^+^ cell motility changed significantly. Although the number of moving CX3CR1^+^ cells was significantly lower in both groups compared with 4 days after cell inoculation, the velocity of movement was significantly increased in E0771-mCherry-sGRP78-bearing mice (102.9 ± 18.28 μm/min). In contrast, in E0771-mCherry-bearing mice, the velocity of movement did not differ significantly between 4 and 7 days after cell inoculation (6.346 ± 0.7385; *n* = 46 cells from 4 to 5 mice; Figure 4E). We found no significant differences in the migration trajectories, and arrest coefficient between 4 and 7 days after cell inoculation, suggesting that the effect of sGRP78 was reduced and that CX3CR1^+^ cells were saturated in sGRP78. These data suggest an association between the level of tumor-secreted GRP78 and the level of CX3CR1^+^ cell migration in metastatic lesions, particularly during the early stages of cancer cell colonization. The effect of sGRP78 on CX3CR1^+^ cell motility was less profound 7 days after cell inoculation, suggesting that sGRP78 promotes immune tolerance early during pre-metastatic niche establishment by recruiting CX3CR1^+^ cells.

### Tumor-Secreted GRP78 Inhibits Hepatic NK Cell Activation

**FIGURE 5:**
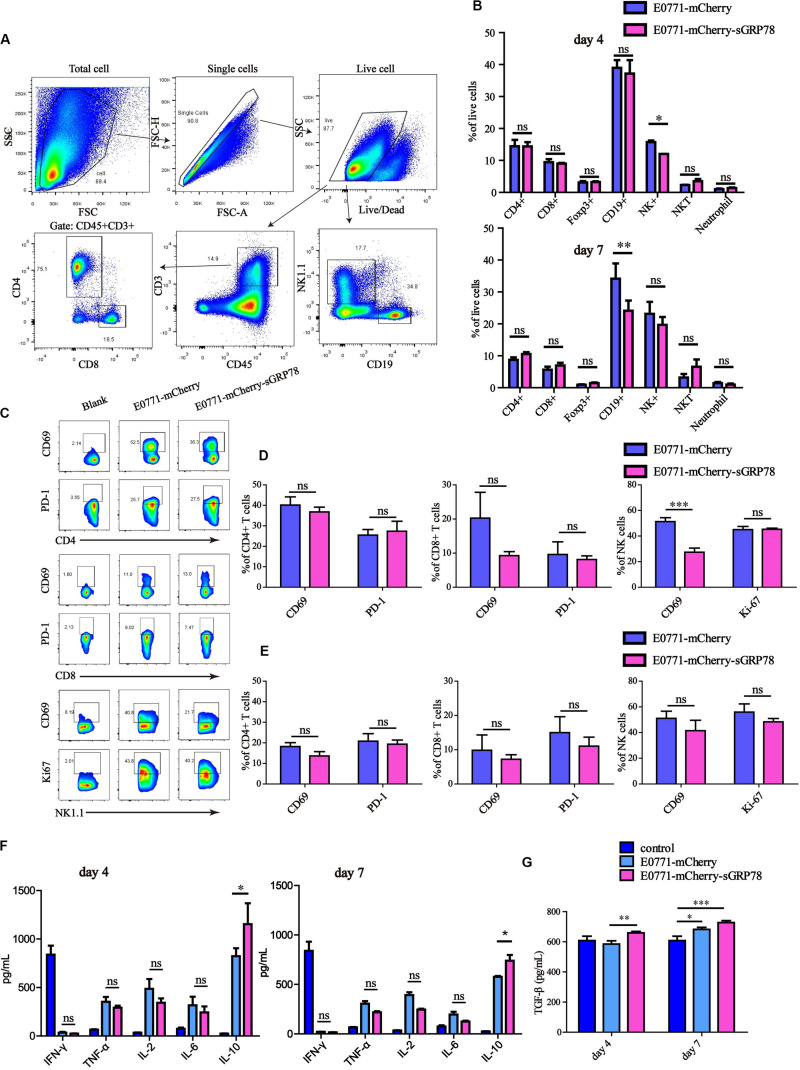
Tumor-secreted GRP78 inhibits hepatic NK cell activation. Liver tissues were excised and their mass was measured at days 4 and 7 after injection. The liver was digested and then filtered. The cell pellet containing leukocytes was collected by Percoll centrifugation. Then cells were stained with antibodies for FCM analysis. **(A)** Gating strategy followed to distinguish the hepatic immune cells. **(B)** Frequencies of immune cell subsets in the liver four (upper panel) and seven (lower panel) days after tumor cell intrasplenic injection. **(C–E)** Frequencies of CD69^+^ and PD-L1^+^ cells among CD4, CD8 subsets, as well as of CD69^+^ and Ki-67^+^ cells in NK1.1 cells. Representative flow cytometry dot plots **(C)** and percentage of positive cells **(D,E)** are shown. **(F,G)** Detection of cytokines. Graphs represent the mean ± SEM. Ns, not significant; ^∗^*P* < 0.05; ^∗∗^*P* < 0.01; ^∗∗∗^*P* < 0.001. Statistical significance was determined by Student’s *t*-test **(B,D,E)** or by Welch’s ANOVA **(F,G)**.

NK cells play essential roles in anti-tumor immunity by exerting cytotoxic effects on cancer cells. Hence, we tested the effects of sGRP78 overexpression on NK cells and other effector lymphocytes, including natural killer T (NKT) cells, B cells, CD4^+^, and CD8^+^ T cells, as well as on macrophages and neutrophils, during liver metastasis (Figure 5A). The frequencies of infiltrated NK (*P* < 0.05) and CD19^+^ B cells (*P* < 0.01) in E0771-mCherry-sGRP78-bearing mice 4 and 7 days after cell inoculation were lower than those in control mice, respectively (Figure 5B). Four days after cell inoculation, the expression of CD69, which is an important marker for activated effector cells (41, 42), was lower on NK cells in sGRP78-expressing mice than control mice (*P* < 0.001; Figures 5C,D). However, no differences in CD69 expression were observed 7 days after cell inoculation (Figure 5E). T cells, B cells, and other lymphocytes were not influenced by sGRP78. The effects of sGRP78 on the recruitment and activation of immune cells was reflected on cytokines secretion. Compared with control mice, sGRP78-expressing mice exhibited higher levels of IL-10 at both time points. Nevertheless, no differences were observed on the levels of IFN-γ, TNF-α, IL-2, or IL-6 (Figure 5F). Notably, compared with control mice, TGF-β levels were increased by 1.12-fold in sGRP78-expressing mice; however, TGF-β upregulation was not observed before day 7 (Figure 5G). These results suggest that tumor-secreted GRP78 facilitates tumor metastasis by inhibiting NK activity and inducing the secretion of immune-suppressive cytokines.

## Discussion

**FIGURE 6:**
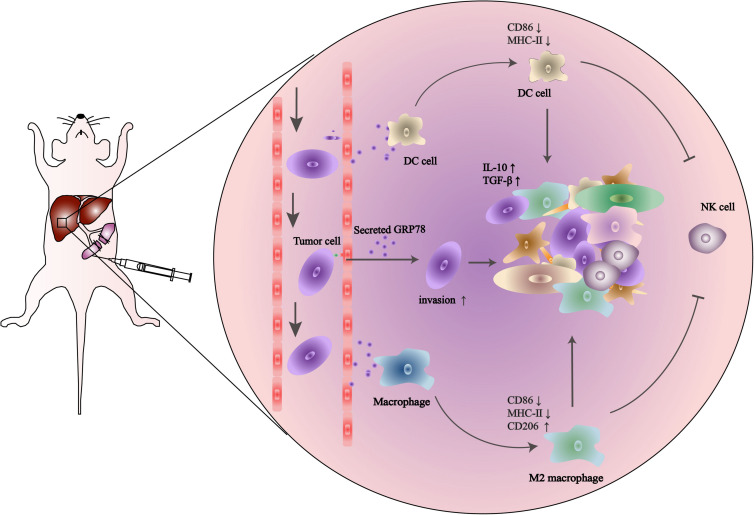
Schematic description of the role of tumor-secreted GRP78 in the pre-metastatic niche. Tumor-secreted GRP78 interacts with hepatic DCs and macrophages in the metastatic niche to induce tolerogenic phenotypes. These impair NK cell recruitment and activation, thereby promoting the establishment of a pre-metastatic niche, which fosters tumor cell colonization, invasion, and metastasis.

In this study, we demonstrated that high expression of sGRP78 in the liver promotes immune tolerance. To elucidate the role of sGRP78 in the hepatic pre-metastatic niche, we established an experimental liver metastasis model using sGRP78-overexpressing murine breast cancer cells. We found that tumor-secreted GRP78 reshaping the hepatic microenvironment by recruiting CX3CR1^+^ myeloid cells. sGRP78 inhibited DC activation by downregulating MHC-II, as well as induced F4/80^+^ M2-type polarization. Furthermore, sGRP78 affected NK cell infiltration and activation in hepatic metastatic lesions by regulating cytokine production (Figure 6).

GRP78 is expressed at higher levels in tumor tissues compared to adjacent healthy tissues, indicating that GRP78 has potential pro-tumorigenic functions (43–45). In order to investigate the effects of sGRP78 on the establishment of the hepatic pre-metastatic niche, we assessed the concentrations of GRP78 in different tissues. GRP78 was highly expressed in the liver, suggesting a potential role in reshaping the hepatic microenvironment to facilitate liver metastasis. sGRP78 has been implicated in immunomodulation by biding to pattern recognition receptors (9, 18). Moreover, intracellular GRP78 levels have been associated with drug resistance and apoptosis in cancers (11, 46). To assess the ability of sGRP78 to reshape the hepatic microenvironment, we established a sGRP78-overexpressing breast cancer cell line (E0771-mCherry-sGRP78) and found that sGRP78 overexpression promoted tumor cell invasion *in vitro*. In addition, sGRP78 overexpression enhanced the colonization and proliferation of metastatic cells in the liver, suggesting that sGRP78 expression in cancer cells promotes invasion and liver metastasis by modulating the hepatic microenvironment.

The establishment of a pre-metastatic niche is a prerequisite for tumor cell colonization and growth in secondary organs (1). In this study, we found increased numbers of tumor cells in the liver 4 days after cell inoculation when sGRP78 was overexpressed, suggesting that the high levels of GRP78 contributed to the establishment of immune tolerance and pre-metastatic niche in the liver. The mechanisms underlying the sGRP78-mediated immune cell recruitment into the liver during the establishment of the pre-metastatic niche merits further investigation. Zhuoyu Li et al. (47) proposed that tumor-secreted GRP78 facilitates macrophage infiltration into the tumor by binding to intracellular Ca^2+^, leading to cytoskeleton remodeling. Our results supported the role of sGRP78 on the motility of CX3CR1^+^ cells in the liver, including DCs and macrophages, fostering liver metastasis. We believe that the increased infiltration of DCs and macrophages accompanied by the decreased recruitment and activation of NK cells contributed to the establishment of a microenvironment allowing for tumor cell survival and growth. Nevertheless, future studies are required to determine the mechanisms involved in the sGRP78-mediated NK cell inhibition.

In conclusion, we have demonstrated that sGRP78 promotes tumor cell colonization in the liver. We also identified several mechanisms involved in this phenomenon, including the modulation of DCs and F4/80^+^ macrophages, induction of TGF-β, and suppression of hepatic NK cells. Thus, sGRP78 promotes tumor growth and metastasis by remodeling the tumor microenvironment and promoting immune tolerance. The ability of GRP78-targeting agents to prevent liver metastasis should be further investigated.

## Data Availability Statement

The raw data supporting the conclusions of this article will be made available by the authors, without undue reservation.

## Ethics Statement

The animal study was reviewed and approved by the Animal Experimentation Ethics Committee of Huazhong University of Science and Technology.

## Author Contributions

LC under the direction of ZZ and GS designed and performed the experiments, analyzed the data, and wrote the first version of the manuscript. XY and LL provided technical guidance. HZhe discussed the manuscript and provided feedback and suggestions. ZZ, PL, and GS designed the experiments, wrote the manuscript, and supervised the study. All authors contributed to the critical revision of the manuscript and approved the final version.

## Conflict of Interest

The authors declare that the research was conducted in the absence of any commercial or financial relationships that could be construed as a potential conflict of interest.

## Supplementary Material

Supplementary Figure 1 GRP78 expression in different mouse organs. GRP78 expression in different tissue homogenates was detected by ELISA. Error bars represent SEM. ***P* < 0.01; ****P* < 0.001. Statistical significance was determined by Welch’s ANOVA.

Supplementary Figure 2 Tumor-secreted GRP78 promotes liver metastasis. **(A)** The photo image of livers (*n* = 5 animals/group) were mice sacrificed on day 20 and day 14 after injected with 1 × 10^6^ E0771 or 5 × 10^5^ B16F10 cells, respectively. **(B)** Representative images of H&E-stained liver sections from mice sacrificed 14 days after tumor cell intrasplenic injection. Scale bar, 2,000 μm. **(C)** Quantification of liver weight (left panel) and metastatic burden (right panel) in livers (*n* = 5 mice per group). **(D)** Survival curves (*n* = 6). Error bars represent SEM. Ns, not significant; **P* < 0.05; ***P* < 0.01; ****P* < 0.001. Statistical significance was determined by Welch’s ANOVA **(C)** or by log-rank Mantel-Cox test **(D)**.
