# Supplementary figures and images for: Tumor-Secreted GRP78 Promotes the Establishment of a Pre-metastatic Niche in the Liver Microenvironment
Source: Front Immunol. 2020 Sep 29;11:584458. doi: 10.3389/fimmu.2020.584458 (PMC7550426; doi:10.3389/fimmu.2020.584458)

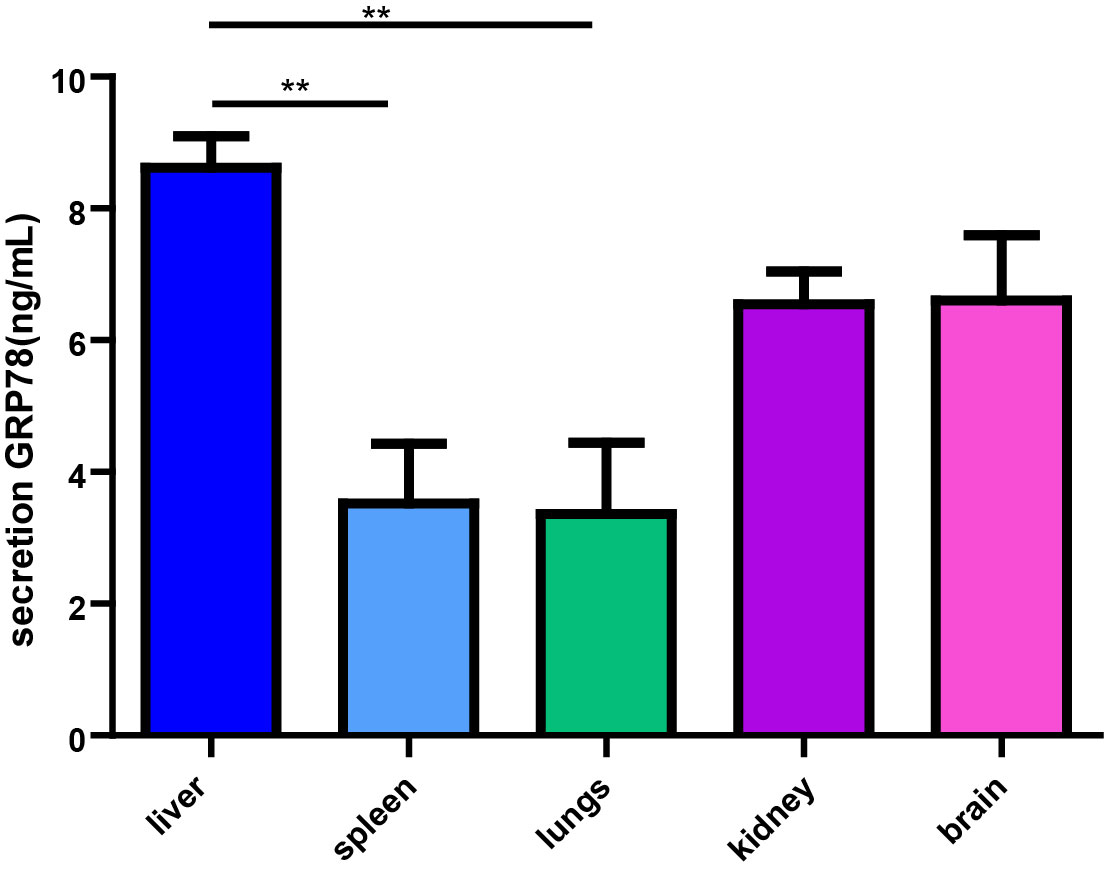

Supplement: Supplementary Figure 1 — GRP78 expression in different mouse organs. GRP78 expression in different tissue homogenates was detected by ELISA. Error bars represent SEM. **P < 0.01; ***P < 0.001. Statistical significance was determined by Welch’s ANOVA. [file Image_1.jpeg]

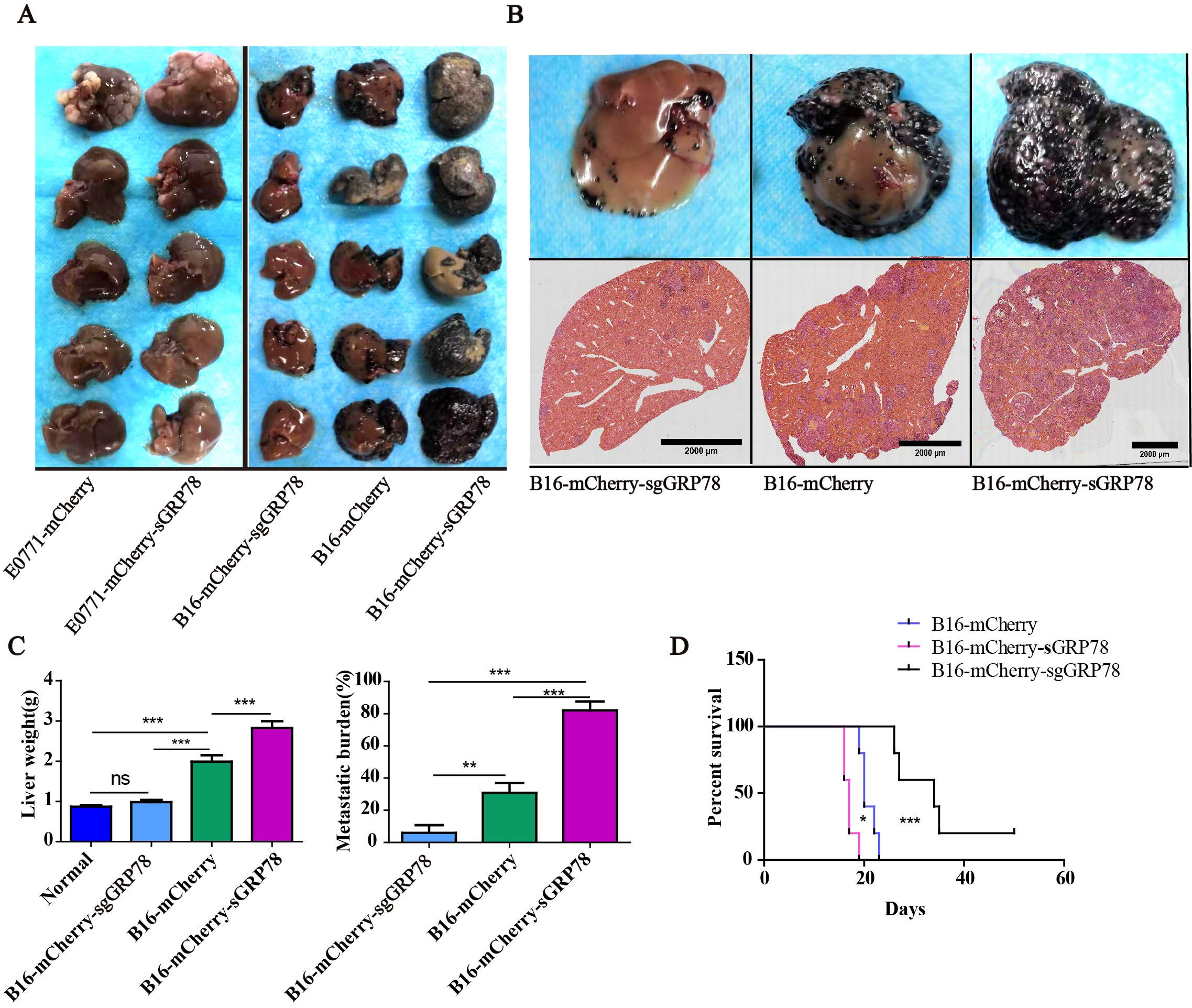

Supplement: Supplementary Figure 2 — Tumor-secreted GRP78 promotes liver metastasis. (A) The photo image of livers (n = 5 animals/group) were mice sacrificed on day 20 and day 14 after injected with 1 × 10^6^ E0771 or 5 × 10^5^ B16F10 cells, respectively. (B) Representative images of H&E-stained liver sections from mice sacrificed 14 days after tumor cell intrasplenic injection. Scale bar, 2,000 μm. (C) Quantification of liver weight (left panel) and metastatic burden (right panel) in livers (n = 5 mice per group). (D) Survival curves (n = 6). Error bars represent SEM. Ns, not significant; *P < 0.05; **P < 0.01; ***P < 0.001. Statistical significance was determined by Welch’s ANOVA (C) or by log-rank Mantel-Cox test (D). [file Image_2.jpeg]
